# AXL Inhibition Extinguishes Primitive JAK2 Mutated Myeloproliferative Neoplasm Progenitor Cells

**DOI:** 10.1097/HS9.0000000000000233

**Published:** 2019-04-24

**Authors:** Stella Pearson, Rognvald Blance, Tim C.P. Somervaille, Anthony D. Whetton, Andrew Pierce

**Affiliations:** 1 Stem Cell and Leukaemia Proteomics Laboratory, Manchester Academic Health Science Centre, The University of Manchester, UK; 2 Leukaemia Biology Laboratory, CRUK Manchester Institute, Manchester, UK; 3 Stoller Biomarker Discovery Centre, The University of Manchester, UK.

## Abstract

Myeloproliferative neoplasms (MPN) are clonal stem cell associated disorders inclusive of chronic myeloid leukemia (CML), Polycythaemia vera (PV), myelofibrosis (MF), and essential thrombocythemia (ET). They are characterized by increased production of myeloid cells with minimal effects on terminal differentiation but can undergo transformation to acute leukemias. PV is the most common chronic myeloproliferative neoplasm and in the majority of cases is characterized by a V617F point mutation in JAK2. This JAK2 activating mutation is also found in about half the patients with MF and ET. Such aberrant proteins offer great potential for the treatment of these diseases however inhibitors to JAK2 have had limited success in the clinic in terms of curing the disease. We have previously used advanced proteomic techniques to identify drug targets and thus develop novel treatment strategies to distinguish the leukemic clone in both CML and PV. Here, we build on our proteomic data sets to characterize a new target, the receptor tyrosine kinase AXL. AXL is overexpressed in acute myeloid leukemia and importantly small molecule inhibitors have been developed which are currently in clinical trial hence offer the opportunity to repurpose this drug for the treatment of MPNs. We demonstrate that AXL is upregulated and activated in JAK2 associated MPNs. Further we show that inhibition of AXL preferentially kills early hemopoietic stem cells from PV patients and as such represents a promising therapeutic approach for JAK2 driven MPNs.

## Abstract 2

Supplemental Digital Content is available in the text

## Introduction

The myeloproliferative neoplasms (MPN) are hemopoietic stem cell disorders characterized by increased proliferation of erythroid, megakaryocytic, or granulocytic cells with minimal effects on terminal differentiation which leads to clinical features such as splenomegaly, thrombosis and hemorrhage.^1^ Some patients also undergo transformation to an acute leukemia. The MPNs include chronic myeloid leukemia (CML), polycythemia vera (PV), myelofibrosis (MF), and essential thrombocythemia (ET). CML is defined by the presence of the activated protein tyrosine kinase (PTK) BCR/ABL^2^ whilst over 90% of patients with PV, and about half of patients presenting with MF and ET have the activating JAK2 V617F PTK mutation.^3–6^ The introduction of inhibitors targeting these leukemogenic PTKs has seen major improvements in treatment especially in CML where the BCR/ABL PTK is targeted.^7,8^ However, PTK inhibitors often fail to induce durable cytogenetic and molecular responses and are rarely curative due to the persistence of leukemic stem cells which can, and do, develop resistance to the drugs employed.^9,10^ We have previously used proteomics to identify MPN stem cell drug targets other than the BCR/ABL^11^ and JAK2 V617F^12^ oncoproteins, to eradicate the diseases as opposed to managing them (as is seen with PTK inhibitors). Despite differing hallmark oncogenes we have shown a degree of similarity between the proteomic perturbations observed in CML and PV and that dual targeting of p53 and MYC is successful in eradicating the leukemic stem cell in both BCR/ABL and JAK2 associated MPNs.^11,12^ Here, we describe another such outcome from our proteomic screens and demonstrate that inhibition of AXL represents a novel therapeutic approach in JAK2 induced MPNs suitable for evaluation in clinical trials. AXL is a receptor protein tyrosine kinase whose ligand, Growth Arrest Specific 6 (GAS6) mediates intracellular signaling via the PI3K/AKT, ERK and PLC pathways, affecting diverse cellular functions including enhanced cell survival and proliferation.^13^ AXL overexpression contributes to drug resistance in several cancers including non-small cell lung carcinoma^14^ and acute myeloid leukemia (AML).^15^ BGB324 is a well-tolerated selective small molecule inhibitor of AXL already in clinical trials as a single agent for AML and in a drug combination for lung cancer treatment (eg, Clinicaltrials.gov identifiers NCT02488408, NCT02424617). Thus, BGB324 offers exciting opportunities for repurposing for the treatment of MPNs circumventing issues relating to drug safety, pharmacokinetics and clinical activity.

## Results and discussion

AXL and its ligand, GAS6, play a critical role in erythropoiesis^16,17^ a process that is disrupted in PV. AXL has recently been demonstrated to be involved in drug resistance in CML and its inhibition shown to have therapeutic potential in BCR/ABL drug-resistant CML.^18,19^ The role of AXL in drug resistance in CML has been linked to increases in LYN kinase activity.^18,20,21^ Given these facts and our previous work demonstrating the similarity between the proteomic perturbations observed in CML and PV^11,12^ and our observation that LYN kinase is upregulated by JAK2 V617F^12^ and MPL W515L^22^ we evaluated AXL as a candidate drug target in MPN.

**Figure 1:**
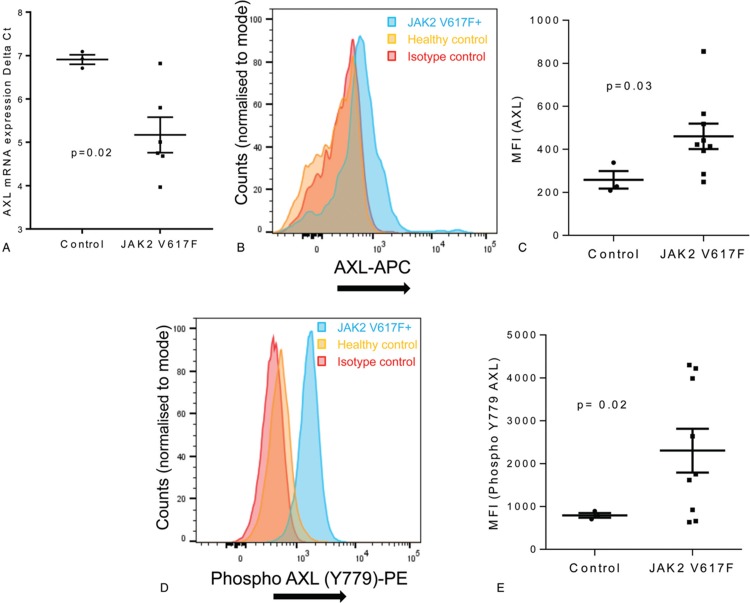
**AXL is upregulated and activated in MPN**; CD34^+^ cells were enriched from control and JAK2 V617F patients using CliniMACS (Miltenyi Biotec). mRNA expression levels for *AXL* (A) in CD34^+^ cells from control and JAK2 V617F patients were measured by qRT-PCR and expressed as delta ct values (mean ± SEM, n = 3 normals, n = 6 for JAK2 V617F). CD34^+^ cells isolated from normal and JAK2 V617F patients were stained with anti AXL (B,C) and anti Y779 phospho-AXL (D,E) antibody and expression levels analyzed on a Novocyte flow cytometer (ACEA Biosciences) using FloJo software. Representative FACS plots are shown (B and D) and amalgamated data (C and E) displayed as median fluorescent intensity ± SEM (n = 3 normals, n = 9 for JAK2 V617F). The results of a *t* test are shown.

**Figure 2:**
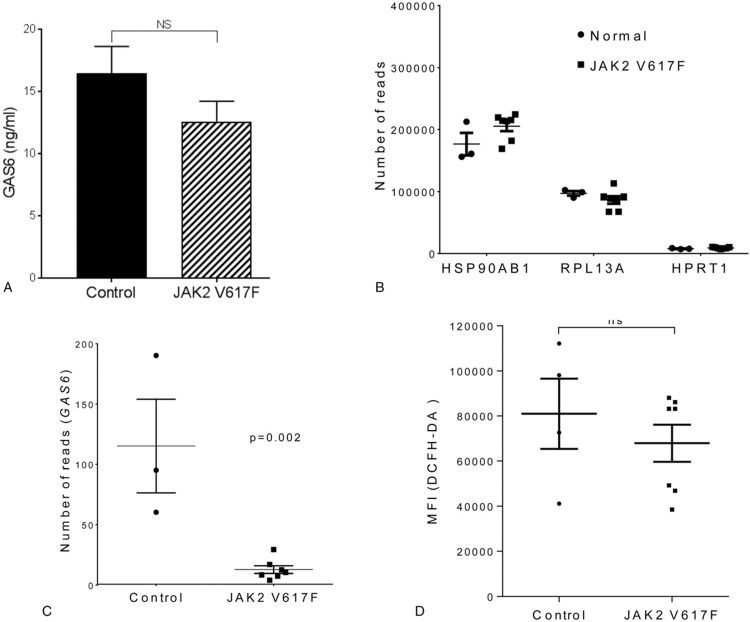
**GAS6 levels in MPN**; A, Levels of GAS6 were measured in plasma of normal and JAK2 V617F patients by ELISA and the results expressed as ng/ml of plasma (mean ± SEM, n = 6). RNA expression levels in CD34^+^ cells were measured using Illumina HiSeq NGS and data shown as the number of normalized RNA reads observed. Results for housekeeping genes (B) and GAS6 (C) are shown (mean ± SEM, n = 3 normals, n = 7 for JAK2 V617F). D, CD34^+^ cells isolated from MPN patients and healthy controls were incubated with 20 μM DCFDA to assess ROS levels. Amalgamated flow cytometry data is displayed as median fluorescent intensity ± SEM (n = 3 normals, n = 7 for JAK2 V617F). The results of a *t* test are shown, ns indicates not significant.

Firstly we investigated *AXL* mRNA levels and found a significant increase (3.2 ± 0.46 fold increase ± SEM) in expression in primary CD34^+^ cells recovered from the blood of patients with MPN compared to cells isolated from healthy controls (Fig. 1A). This was reflected by an increase in protein expression of both AXL and tyrosine 779 phosphorylated AXL, as assessed by flow cytometry (Fig. 1B–E, see also supplementary Figure 1, Supplemental Digital Content). It has been reported that the increase in AXL phosphorylation observed in CML^18^ is a consequence of increases in its ligand GAS6. We, therefore, measured the levels of GAS6 in plasma by ELISA. Although AXL expression is elevated and AXL displays increased activation in CD34^+^ cells from MPN patients this was not due to changes in the levels of circulating GAS6 which appear equivalent in MPN and control patients (Fig. 2A). Worthy of note is the fact that GAS6 mRNA levels are actually decreased in CD34^+^ cells from MPN patients (Fig. 2B-C) presumably as a consequence of a negative feed-back loop due to the activated AXL seen in these cells (Fig. 1E). It is perhaps not surprising that this decrease in CD34^+^ cell production of GAS6 mRNA is not reflected in reduced circulating GAS6 as these cells are unlikely to be the major source of GAS6 in the blood stream. In fact it has been reported that the elevated circulating GAS6 levels in AML^15^ and CML^19^ are due to an increased production by the bone marrow derived stromal cells which suggest a role for paracrine signaling between leukemia cells and the bone marrow microenvironment. GAS6-independent AXL phosphorylation has been reported previously via homophilic binding as a consequence of overexpression^23^ and/or due to the presence of reactive oxygen species.^24^ Ligand independent AXL activation as a consequence of oxidative stress is perhaps particularly pertinent given the role of reactive oxygen species in JAK2 V617F disease progression,^25^ however, we were unable to detect differences in intracellular ROS levels between CD34^+^ cells from MPN and control patients (Fig. 2D). This does not rule out a role for ROS in the observed activation of AXL as extracellular levels of ROS have been shown to activate AXL.^24^ An alternative explanation could be an increase in circulating phosphatidylserine. MPN patients are characterized by increased circulating microparticles and phosphatidylserine exposing erythrocytes^26^ and it has been reported that AXL activation is markedly increased in the presence of phosphatidylserine.^27,28^

**Figure 3:**
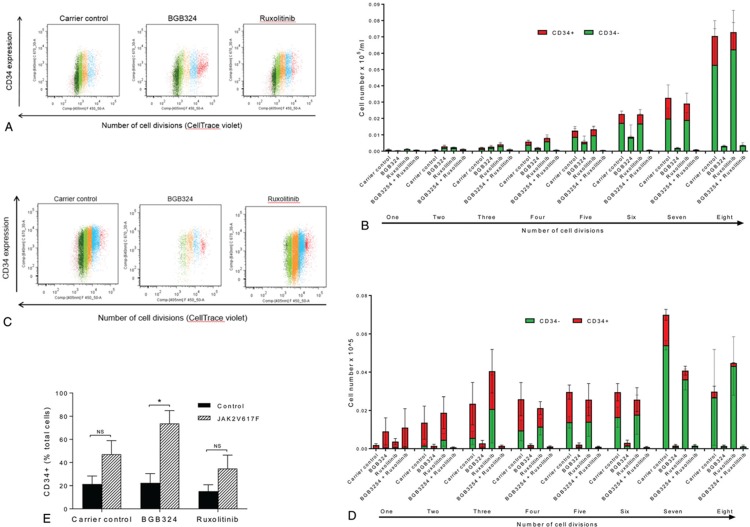
**BGB324 has distinct effects upon differentiation in normal and JAK2 V617F expressing cells**; The effect of inhibition of AXL (3 μM BGB324) and JAK2 (50 nM Ruxolitinib) on cell proliferation and differentiation of CD34^+^ cells enriched from control and JAK2 V617F patients were assessed using CellTrace Violet Cell Proliferation Kit (Molecular Probes) and flow cytometry. A, displays a representative experiment for control patient cells and B, data from 4 experiments (mean ± SEM). C, displays a representative experiment for JAK2 V617F positive cells and D, amalgamated data (mean ± SEM, n = 3). E, shows the number of CD34^+^ cells remaining following 8 days in culture expressed as a percentage of the total cell number (calculated from 3b and 3d). The *t* test results are shown or represented by; ns = not significant, ^∗^ < 0.05.

**Figure 4:**
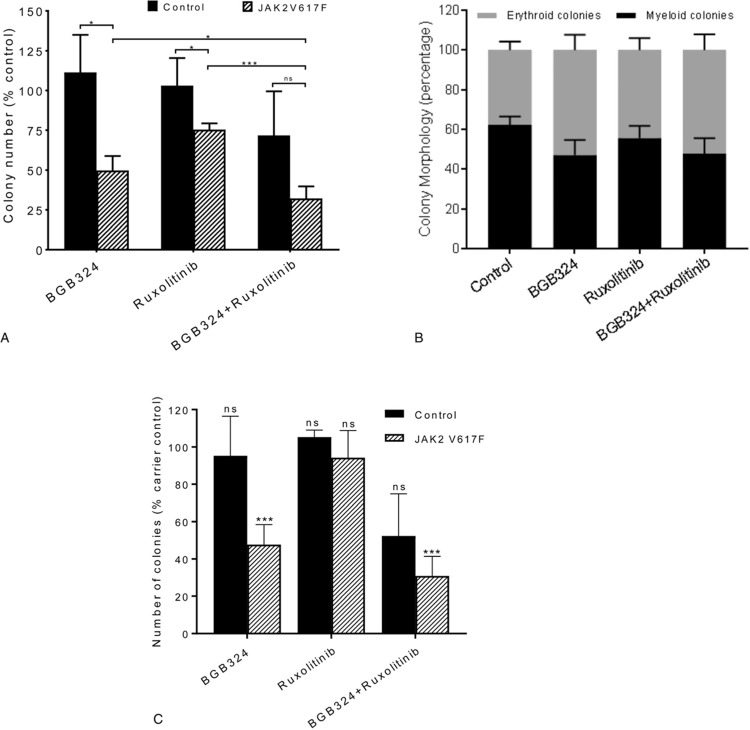
**BGB324 significantly and selectively reduces the clonogenic activity of JAK2 V617F mutant cells**; Colony forming assays were performed with CD34^+^ cells enriched from control and JAK2 V617F patients in the presence and absence of drug (3 μM BGB324 and/or 50 nM Ruxolitinib). Colonies were counted (A) and morphology assessed (B) at day 14. Colony counts are expressed as a percentage of carrier control for each individual patient (mean ± SEM, n = 3 for normals, n = 9 for JAK2 V617F) and morphology displayed as a percentage of colony type (mean ± SEM, n = 9) for JAK2 V617F patient samples. The average number of colonies seen in all carrier control treated samples was 62 ± 14 (mean ± SEM). C; Colonies produced after 7 days were re-suspended and re-plated in methylcellulose in the absence of inhibitors. The number of colonies was assessed following 14 days and the data expressed as a percentage of control for each individual patient (mean ± SEM, n = 3 for control, n = 10 for JAK2 V617F expressing cells). The average number of colonies seen in all carrier control treated samples was 93 ± 20 (mean ± SEM). The results of a *t* test results are represented by; ns = not significant, ^∗^ < 0.05, ^∗∗∗^ < 0.001.

Due to the increased activation of AXL we observed in MPN patients we next investigated the effects of inhibition of AXL on JAK2 V617F driven MPN cells. We chose to use the small molecule inhibitor of AXL, BGB324, as it is in early phase clinical trials in AML and has been shown to preferentially kill drug resistant CML cells.^18^ BGB324 is a highly selective inhibitor of AXL being 50 to 100-fold more selective for AXL than other TAM family members Mer and tyro3.^29^ Preliminary experimental assessment was in line with previous data in CML^18^ and identified 3 μM BGB324 as an effective drug dose differentially inhibiting hemopoietic colony formation from JAK2 V617F positive patients compared to those from healthy controls (Supplementary Figure 2, Supplemental Digital Content). BGB324 was also shown to inhibit AXL phosphorylation in CD34^+^ cells isolated from patients harbouring the JAK2 V617F mutation at this dose (Supplementary Figure 3, Supplemental Digital Content). This concentration of BGB324 has also been used widely in other diseases and has been shown to be achievable in plasma in vivo in both animal models^29^ and phase 1 clinical trials.^30^ In preclinical studies in mice a single dose of BGB324 at 75 mg/kg resulted in a C_max_ of 6.8 μM with a plasma half-life of 13 hours.^29^ The clinically prescribed JAK2 inhibitor ruxolitinib was included for comparison and to investigate any potential benefit from a dual treatment approach. Initial experiments were undertaken on CD34^+^ cells in liquid culture labelled with CellTrace™ to allow analysis of both cell division and differentiation. Ruxolitinib had very little effect on the proliferation of either control or JAK2 V617F expressing cells. BGB324, alone and in combination with ruxolitinib, substantially reduced the proliferation of both control (Fig. 3A,B) and JAK2 V617F expressing CD34^+^ cells (Fig. 3C,D) when compared to untreated and ruxolitinib-treated cells. However, BGB324 did have a differential effect on control and MPN patient samples in terms of CD34^+^ expression levels (Fig. 3E). This indicates that BGB324 has distinct effects upon differentiation in normal and JAK2 V617F expressing cells which we investigated further using colony forming assays on CD34^+^ cells isolated from both JAK2 V617F positive patients and non-diseased controls in the presence and absence of ruxolitinib and BGB324 (Fig. 4A). Inhibition of AXL with BGB324 significantly and selectively reduced the clonogenic activity of CD34^+^ cells isolated from patients expressing JAK2 V617F but not that of normal CD34^+^ cells, and to an extent greater than that observed with ruxolitinib alone. The combination of ruxolitinib and BGB324 was more effective than BGB324 alone in inhibiting the clonogenic activity of MPN patient CD34^+^ cells, but also reduced the clonogenic activity of normal CD34^+^ cells (Fig. 4A). This is in keeping with previous data where despite ruxolitinib having FDA approval for use in MPN its differential effect on normal and PV cells in vitro has only been demonstrated in the absence of erythropoietin.^31^ Furthermore, the response of mutant JAK2 acute lymphoblastic leukemia xenografts to ruxolitinib is variable^32^ highlighting the need for new treatment strategies other than JAK2 inhibitors.

**Figure 5:**
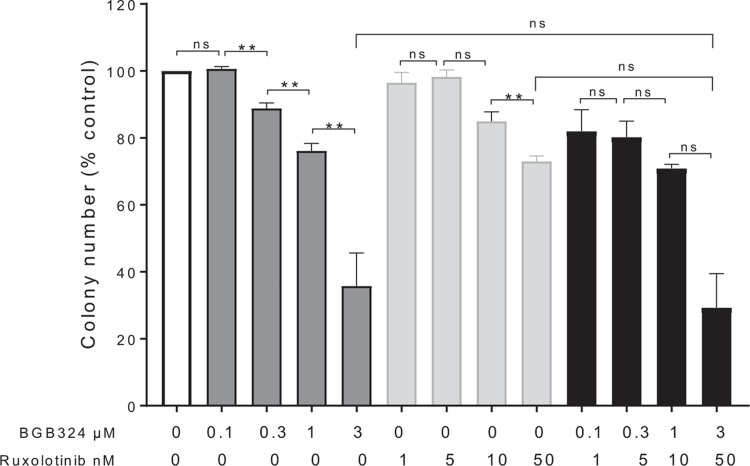
**BGB324 and ruxolitinib do not act synergistically**; A, Colony forming assays were performed with CD34^+^ cells enriched from JAK2 V617F patients. The colony forming assays were performed in the absence or presence of BGB324 and ruxolitinib as a single agent or in combination at the concentrations shown. The number of colonies produced after 14 days were counted and expressed as a percentage of carrier control for each patient (mean ± SEM, n = 3). The average number of colonies seen in carrier control was 100 ± 3 (mean ± SEM). The results of a *t* test results are represented by; ns = not significant, ^∗∗^ < 0.01.

We were unable to detect an effect on colony morphology (Fig. 4B) however, the colonies produced in the presence of BGB324 displayed a reduced replating capacity (Fig. 4C). This suggests that BGB324 has a selective effect on MPN patient primitive CD34^+^ colony forming cells inferring an ability to extinguish the preleukemic clone. All patients tested were 100% mutant for JAK2 V617F in the circulating CD34^+^ compartment, precluding any direct measurement on specificity, and as such may represent a subset of clonally dominant MPNs. To investigate the lack of synergy between BGB324 and ruxolitinib we performed colony forming assays with JAK2 V617F expressing CD34^+^ cells from patients with MPN with a range of drug doses alone and in combination (Fig. 5A). Whilst we saw an increase in inhibition of colony formation with increasing doses of both BGB324 and ruxolitinib alone we failed to find a drug dose combination that led to a synergistic inhibition of colony formation. This suggests the 2 drugs may be affecting the same pathway. There is evidence for this in the literature, for example, AXL inhibition has been reported to exert its anti-tumor effects through AKT/PI3K signaling^15,33^ a pathway known to be activated in MPN.^4^ Furthermore, AXL family members have been shown to lead to the phosphorylation of JAK2 target STAT family kinases in AML and ALL cells.^34,35^ Also, other receptor tyrosine kinases are known to activate JAK/STAT signaling with activation of PDGFR resulting in the JAK-independent tyrosine phosphorylation of STATs.^36,37^ This is presumed to be via Src kinase which is of particular relevance given our observation on the upregulation of the src family member LYN in MPN. In addition, the AXL regulated Ras pathway causes the activation of MAPK which is capable of phosphorylating STAT on serine which whilst not absolutely necessary for STAT activity dramatically enhances transcriptional activation by STAT.^38^

**Figure 6:**
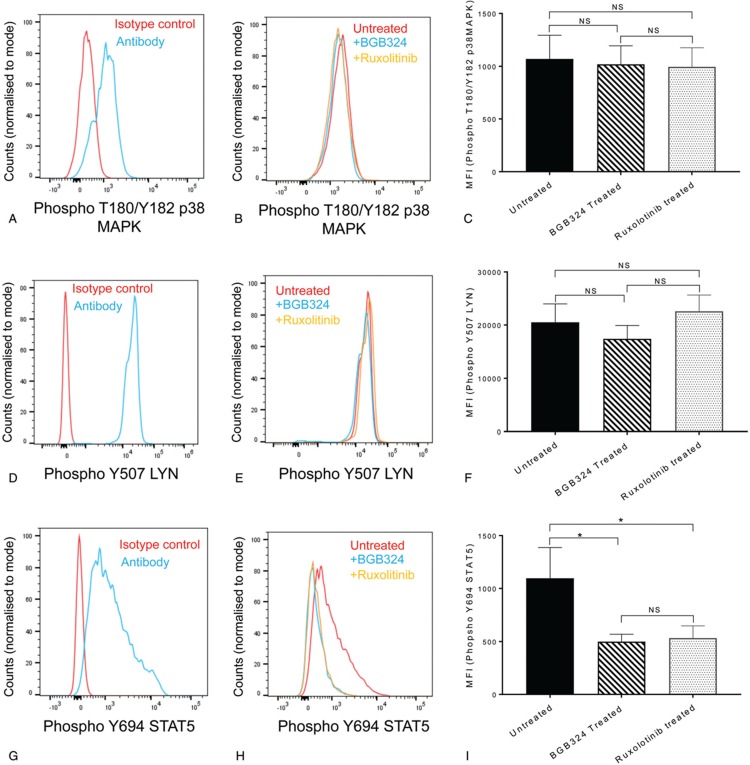
**BGB324 and ruxolitinib reduce STAT5 phosphorylation observed in PV patients**; CD34^+^ cells isolated from JAK2 V617F patients were cultured in Fischer medium supplemented with 5% horse serum in the presence or absence of 3 μM BGB324 or 50 nM ruxolotinib for 4 hours and the effects on T180/Y182 phospho-p38MAPK (A–C), Y507 phospho-LYN (D–F) and Y694 phospho-STAT5a (G–I) analyzed on a Novocyte flow cytometer (ACEA Biosciences) using FloJo software. Representative FACS plots are shown (A, B, D, E, G, and H) and amalgamated data (C, F, and I) displayed as median fluorescent intensity ± SEM (n = 9 for STAT5, n = 5 for p38MAPK and LYN). The results of a *t* test are shown (NS = not significant, ^∗^ < 0.05).

To investigate the potential interaction between AXL induced signaling and JAK2 activation in MPN we assessed the phosphorylation status of STAT5, ERK, AKT, LYN, and p38MAPK (Supplementary Figure 4, Supplemental Digital Content) as surrogate markers of activation and the effects of AXL and JAK2 inhibition on these phosphorylation events. We were unable to detect ERK or AKT phosphorylation in CD34^+^ cells isolated from MPN patients (Supplementary Figure 5, Supplemental Digital Content). We were able to detect phosphorylation of T180/Y182 of p38MAPK (Fig. 6A–C) and Y507 of LYN (Fig. 6D–F) but neither were affected by BGB324 or ruxoloitinib treatment over a 4 hour period (Fig. 6C and F). In contrast, the STAT5a phosphorylation we observed in MPN patients (Fig. 6 G–I) was reduced by both AXL inhibition with BGB324 and JAK2 inhibition with ruxolitinib (Fig. 6H and I). Thus, AXL and JAK signaling overlap to some degree. This helps explains the lack of synergy between BGB324 and ruxolitinib but does not answer why BGB324 is more effective in inhibiting CD34^+^ cells colony formation. Since, AXL activation plays a role in the resistance to targeted and cytotoxic therapies of several different tumors where receptor tyrosine kinases are involved, for example, AML resistance to FLT3 tyrosine kinase inhibitors,^39^ HER-2 positive breast cancer^40^ and rhabdomyosarcoma resistant to IGF1R inhibition^41^ a deeper understanding of the complex interaction between AXL signaling and other pleiotropic signaling events is still required to fully understand the lack of synergy between AXL and JAK2 targeted inhibitors. In addition, since we did not evaluate JAK2-negative MPN patients with other driver mutations, it remains unclear whether or not AXL inhibition is specific to JAK2-mutant MPNs.

In conclusion, we have identified a molecular perturbation shared in MPN and CML that is a potential therapeutic target in MPN. Present treatments alleviate the symptom burden but unfortunately fail to cure the disease or prevent transformation. Whilst the introduction of specific protein kinase inhibitors to JAK2, such as ruxolitinib, promised to be a major advance very few patients show reduced allele burden.^10^ As such there is presently an unmet clinical need in PV for better treatments; AXL inhibition represents a novel candidate therapeutic approach in PV suitable for evaluation in clinical trials. BGB324 is a selective and potent small molecule AXL kinase inhibitor in clinical development which is well-tolerated with a favorable safety profile available as an orally administered drug. As such BGB324 offers great opportunities for rapid repurposing for PV treatment.

## Materials and methods

### Patient material

Use of human tissue was in compliance with the ethical and legal framework of the Human Tissue Act. Experiments had ethical approval from the NRES committee of the regional NHS health research authority (14/LO/0489 and 17/LO/0888). Primary human JAK2 V617F positive samples were obtained from the Manchester Cancer Research Centre's Tissue Biobank (instituted with approval of the South Manchester Research Ethics Committee, HTA 30004). Their use was authorized following ethical review by the Tissue Biobank's scientific sub-committee, and with the informed consent of the donor. Patient details are given in Table 1. No differences in response were observed when the patient samples were from those undergoing treatment with ruxolitinib. When ruxolitinib was used in vitro the individual patient data are presented in relation to their pre-treatment in vivo in Supplementary Table 1 and 2 (Supplemental Digital Content). Control samples were cells isolated from Leucocyte cones from patients undergoing leukapheresis within the NHS Blood and Transplant Service for all experiments apart from mRNA measurements were CD34^+^ mobilized cells surplus to requirements from patients undergoing chemotherapy and autologous transplantation for lymphoma or myeloma were used. Their use was authorized by the Salford and Trafford Research Ethics Committee and, for samples collected since 2006, following the written informed consent of donors. The CD34^+^ cell population were enriched using CliniMACS (Miltenyi Biotec) according to standard protocols and previously described.^12^

**Table 1:**
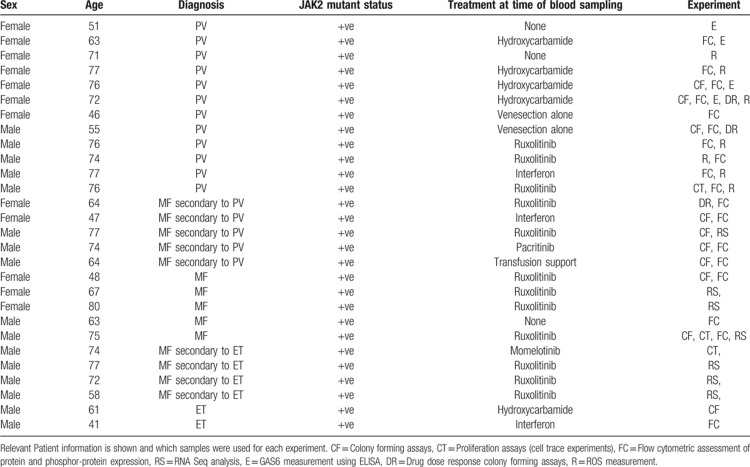
MPN patient data.

### Protein measurements

Antibody details are shown in Supplementary Table 3 (Supplemental Digital Content). Levels of GAS6 were measured using the Human GAS6 Duoset ELISA from R&D Systems (DY885B) as per manufactures instructions. Expression of AXL, Y779 phospho-AXL, Y694 phospho-STAT5, T202/Y204 phospho-ERK1/2, S473 phospho-AKT, Y507 phospho-LYN and T180/Y182 phospho-p38MAPK was assessed using flow cytometry with the Novocyte flow cytometer (ACEA Biosciences) using FloJo software. Briefly, cells were fixed with 1.5% formaldehyde and permeabilized with methanol. Cells were then washed twice with staining medium (PBS, 0.5% BSA, 0.02% sodium azide) before incubation with appropriate antibodies for 30 minutes at room temperature. If required cells were then washed prior to incubation with secondary fluorescent antibody for 30 minutes. Where indicated cells were treated with 3 μM BGB324 or 50 nM ruxolitinib in Fischers medium supplemented with 5% horse serum for 4 hours at 37°C in a 5% CO_2_ incubator.

### RNA measurements

Real-time qPCR assays were designed using Roche Universal ProbeLibrary Assay Design Center. Details of Probes and primer sequences are shown in Supplementary Table 4 (Supplemental Digital Content). Primer efficiencies were determined using cDNA generated from K562 cells and a ten-fold dilution series from 100 pg/μL to 1 fg/μL. Efficiencies (E) were calculated from the gradient of the regression line fitted to a graph of C_q_ vs log cDNA input using the equation E = 10^−1/gradient^. Assays were considered suitable if their efficiencies fell between 95% and 105%. A no-template experiment was run in parallel to control for primer specificity. cDNA was generated from CD34^+^ cells isolated from control and JAK2 V617F positive patient samples. Suitable reference genes were selected using the NormFinder algorithm.^42^ The 2 genes with the highest stability values were found to be SDHA and YWHAZ. Normalization was carried out to the average of these 2 genes. Integrity of all RNA samples was assessed on an Agilent 2100 Bioanalyser. No sample had a RIN value below 8.1. Reverse transcription and cDNA amplification were carried out using Preamp and Reverse Transcription Master Mix Kit, (Fluidigm, 100–6300) according to the manufactures’ instructions using 14 thermal cycles for both. Amplified cDNA was quantified on a Qubit Fluorometer. All samples were randomized and used in qPCR reactions at a concentration of 1 pg/μL. Working concentrations were 900 nM for primers and 250 nM for probes. Taqman gene expression mastermix (Applied Biosystems, #4369016) was diluted 1:1 with sample/primer/probe mix and 10 μL reaction volumes were run in 384-well plates on a Quantstudio 5 real-time PCR system at default settings. Data was analyzed using Quantstudio Design & Analysis software using the 2^−ΔΔCq^ comparative method.^43^

RNA sequence analysis was undertaken on total RNA extracted from CD34^+^ cells isolated from peripheral blood using Qiagen RNeasy Plus kit. RNA sequence data was generated by the Genomic Technologies Core Facility at the University of Manchester. The quality and integrity of total RNA samples were assessed using a 2100 Bioanalyzer (Agilent Technologies) and RNA-seq libraries generated using the TruSeq Stranded mRNA assay (Illumina) according to the manufacturer's protocol. Samples were paired-end sequenced on an Illumina HiSeq2500 instrument. The FastQ files were analyzed with FastQC, and any low-quality reads trimmed with Trimmomatic (www.usadellab.org). All libraries were aligned using Tophat-2 and the mapped reads counted by genes with HTSeq. Raw counts were normalized in DEseq.

### Cell based assays

Cell Trace™ and colony forming assays were performed as previously described.^12^ In brief; CD34^+^ cells were stained using CellTrace Violet Cell Proliferation Kit (Molecular Probes) and cultured in IMDM, 20% (v/v) fetal calf serum, rhIL-3 (20 ng.ml), rhSCF (50ng/ml) and Flt-3 ligand (10ng/ml) (PeproTech). On day zero and following 8 days culture cells were stained with CD34-APC and fluorescence measured on a LSRFortessa™ (Becton Dickenson) flow cytometer. Results were analyzed using FloJo software. CD34^+^ cell colony forming assays were performed in methylcellulose complete media (R&D systems) supplemented with 2u/ml EPO at a density of 3000 cells/ml. To assess retention of self-renewal capacity the resulting colonies at day 7 were replated in methylcellulose and colonies counted at 14 days.

Levels of intracellular reactive oxygen species (ROS) were compared using the cell permeant reagent 2’,7’ –dichlorofluorescin diacetate (DCFDA). CD34^+^ cells isolated from MPN patients and healthy controls were seeded at 1×10^6^/ml in RPMI, 10% FCS and DCFDA added to a final concentration of 20 μM in a final volume of 100 μl. Cells were incubated for 30 minutes at 37°C before pelleting and resuspending in 300 μl of PBS, 10% FCS 0.01% sodium azide. Fluorescence was then measured on a Novocyte flow cytometer with excitation at 488 nm and detection at 535 nm. Results were analyzed using FloJo software.

## Supplementary Material

Supplemental Digital Content
