# Supplementary material for: AXL Inhibition Extinguishes Primitive JAK2 Mutated Myeloproliferative Neoplasm Progenitor Cells
Source: Hemasphere. 2019 Apr 24;3(3):e233. doi: 10.1097/HS9.0000000000000233 (PMC6746025; doi:10.1097/HS9.0000000000000233)
Supplement: Supplemental Digital Content [file hs9-3-e233-s001.pdf]

Supplementary Data

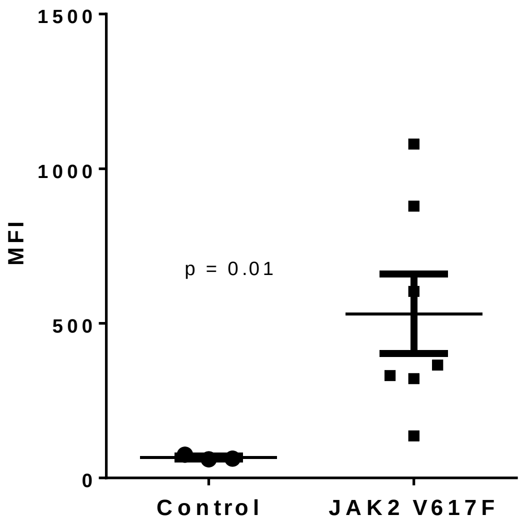

Supplementary Figure 1: Phospho Y779 AXL expression. CD34+ cells isolated from normal (n=3) and JAK2 V617F patients (n=7) were stained with anti Y779 phosphoAXL (R&D systems #IC6965P) and expression levels analysed on a LSR Fortessa flow cytometer (BD Biosciences) using FloJo software. Results are displayed as median fluorescent intensity (MFI) +/- SEM and the results of a statistical t-test are shown.

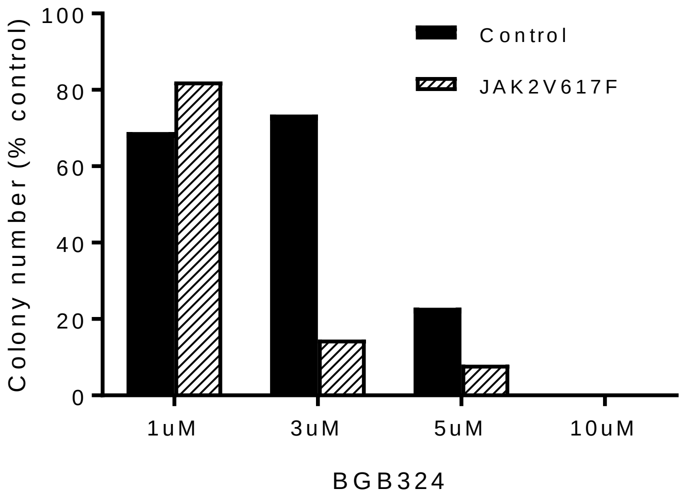

Supplementary Figure 2: Primary cell colony forming assays in the presence of different doses of BGB324. Colony forming assays were performed in the presence of a range of BGB324 doses by plating $CD34^+$ cells in methylcellulose complete media (R&D systems) supplemented with 2u/ml EPO at a density of 3000cells/ml. 0.2ml was dispensed into each of 4 wells of a 24 well plate. Plates were incubated at 37^0C in 5% CO_2 for 14 days before the number of colonies were counted. Data shown for n=1 for control and JAK2V617F PV patient samples

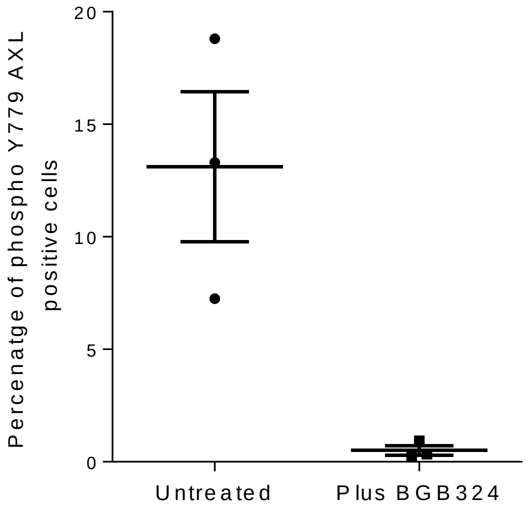

Supplementary Figure 3: Inhibition of phospho Y779 AXL expression by BGB324. CD34+ cells isolated from JAK2 V617F patients were treated with 3µM BGB324 for 4 hours prior to staining with anti Y779 phosphoAXL (R&D systems #IC6965P) and expression levels analysed on a Novocyte flow cytometer (ACEA Biosciences) using FloJo software. Results are displayed as median fluorescent intensity (MFI) +/- SEM n=3.

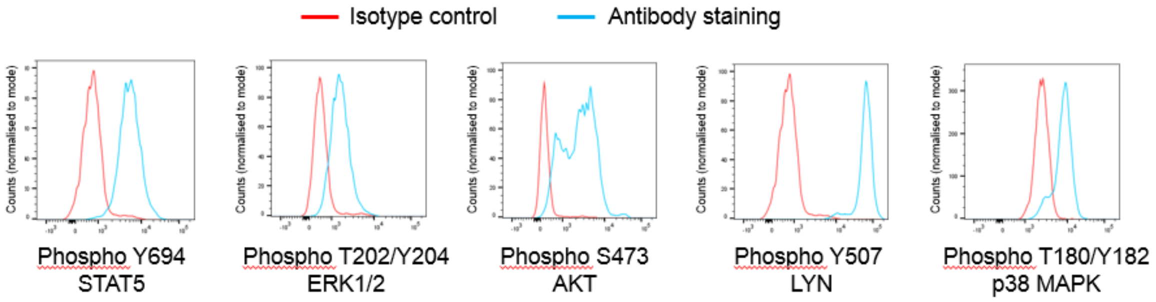

Supplementary Figure 4: Phospho protein detection assay development. JAK2 V617F expressing SET-2 cells were stained with antibodies against Y694 phospho-STAT5, T202/Y204 phospho-ERK1/2, S473 phospho-AKT, Y507 phospho-LYN and T180/Y182 phospho-p38MAPK and expression levels analysed on a Novocyte flow cytometer (ACEA Biosciences) using FloJo software. Representative FACS plots are shown.

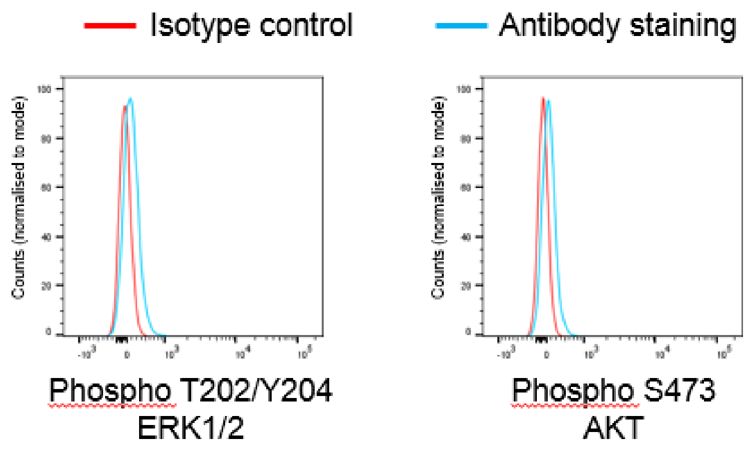

Supplementary Figure 5: Phospho-ERK and phospho-AKT are not detected in CD34+ cells from patients with MPN. CD34+ cells isolated from JAK2 V617F patients were cultured in Fischers medium supplemented with 5% horse serum for 4 hours and then stained with anti T202/Y204 phospho-ERK1/2 and anti S473 phospho-AKT antibody and expression levels analysed on a Novocyte flow cytometer (ACEA Biosciences) using FloJo software. A representative FACS plot from one patient of five examined is shown.

Supplementary Table 1. Colony forming assay data used in Figure 4a

Patient treatment prior to experimentation *in vitro*	Ruxolitinib			Pacritinib	Venesection alone	Transfusion support	Interferon	hydroxycarbamide	
Colony number in untreated sample	47	138	11	32	25	65	131	11	90
Colony number in ruxolitinib treated samples as a percentage of DMSO control	68%	79%	85%	62%	49%	84%	84%	82%	74%

The data shown are the individual data points used in Figure 4a on the effects of ruxolitinib on colony forming ability of $CD34^+$ cells enriched from JAK2 V617F patients. Colony forming assays were performed in the absence or presence of BGB324 and ruxolitinib. The data presented here illustrates the response to ruxolitinib in respect to the treatment the patients had received prior to the *in vitro* experiments. Data includes the number of colonies observed in untreated samples and the colonies observed in the doses of ruxolitinib shown as a percentage of the untreated control.

Supplementary Table 2. Drug dose response data used in Figure 5a

	Ruxolitinib concentration	Patient treatment prior to experimentation *in vitro*		
		Venesection alone	hydroxycarbamide	Ruxolitinib
Colony number in untreated sample	0	94	104	100
Colony number as a percentage of DMSO control	1nM	97%	91%	101%
	5nM	97%	95%	102%
	10nM	79%	89%	87%
	50nM	70%	76%	73%

The data shown are the individual data points used in Figure 5a on the effects of ruxolitinib on colony forming ability of $CD34^+$ cells enriched from JAK2 V617F patients. Colony forming assays were performed in the absence or presence of BGB324 and ruxolitinib as a single agent or in combination at various concentrations. The data presented here illustrates the response to ruxolitinib in respect to the treatment the patients had received prior to the *in vitro* experiments. Data includes the number of colonies observed in untreated samples and the colonies observed in the doses of ruxolitinib shown as a percentage of the untreated control.

Supplementary Table 3: Antibodies used in the study

Antibody	Company (Catalogue number)	Dilution
AXL - APC	R&D systems (FAB154A)	1/10
CD34 - APC	eBioscience (17-0349)	1/200
phospho Y779 AXL - PE	R&D systems (IC6965P)	1/13.3
phospho Y507 LYN	Thermo Fisher (720013)	1/100
phospho T202/Y204 ERK1/2 - AF488	BD bioscience (612592)	1/10
phospho S473 AKT - PE	BD bioscience (560378)	1/10
phospho Y694 STAT5 – AF647	BD bioscience (612599)	1/10
phospho T180/Y182 p38 MAPK – pacific blue	BD bioscience (560313)	1/10

Table detailing the procedures, dilutions and suppliers of the antibodies used in the study

Supplementary Table 4: RT qPCR reagents

Gene	ENSEMBL transcripts	Sequence		Probe and Cat Nº
		Forward	Reverse	
AXL	ENST00000301178.8<br>ENST00000359092.7<br>ENST00000593513.1	gtggctgtgaagacgatgaa	atgcagaccgcttcactca	#65<br>04688643001
SDHA	ENST00000264932.10	cctgtcctatgtggacgttg	gttttgtcgatcacgggtct	# 48<br>04688082001
YWHAZ	ENST00000353245.7	gatccccaatgcttcacaag	tgcttgttgtgactgatcgac	# 30<br>04687639001

Table showing primer sequences used to detect AXL, LYN, SDHA and YWHAZ in RT qPCR experiments. All primers were obtained from Life Technologies (Paisley UK). Probes were obtained from Roche (Burgess Hill, UK) and the probe identifiers and catalogue numbers given. The protein isoforms for the mRNAs detected by the assays used are also displayed.
